# Performance Tiers within a Competitive Age Group of Young Swimmers Are Characterized by Different Kinetic and Kinematic Behaviors

**DOI:** 10.3390/s23115113

**Published:** 2023-05-27

**Authors:** Catarina C. Santos, Nuno D. Garrido, Francisco Cuenca-Fernández, Daniel A. Marinho, Mário J. Costa

**Affiliations:** 1 Department of Sport Sciences, University of Beira Interior, 6201-001 Covilhã, Portugal; catarina.costa.santos@ubi.pt (C.C.S.); dmarinho@ubi.pt (D.A.M.); 2 Research Center in Sports Sciences, Health Sciences and Human Development (CIDESD), 5000-801 Vila Real, Portugal; 3 Department of Sport Sciences, Exercise and Health, University of Trás-os-Montes and Alto Douro, 5000-801 Vila Real, Portugal; 4 Department of Sports and Computer Science, Universidad Pablo de Olavide, 41013 Seville, Spain; fcuefer@upougr.es; 5 Aquatics Lab, Department of Physical Education and Sport, Faculty of Sport Sciences, University of Granada, 18071 Granada, Spain; 6 Centre of Research, Education, Innovation, and Intervention in Sport (CIFI2D), Faculty of Sport, University of Porto, 4200-450 Porto, Portugal; mjcosta@fade.up.pt; 7 Porto Biomechanics Laboratory (LABIOMEP-UP), University of Porto, 4200-450 Porto, Portugal

**Keywords:** swimming, youth, tiers, biomechanics, sensors, force

## Abstract

The present study aimed to analyze swimmers’ in-water kinetic and kinematic behaviors according to different swimming performance tiers within the same age group. An amount of 53 highly trained swimmers (girls and boys: 12.40 ± 0.74 years) were split up into 3 tiers based on their personal best performance (i.e., speed) in the 50 m freestyle event (short-course): lower-tier (1.25 ± 0.08 m·s^−1^); mid-tier (1.45 ± 0.04 m·s^−1^); and top-tier (1.60 ± 0.04 m·s^−1^). The in-water mean peak force was measured during a maximum bout of 25 m front crawl using a differential pressure sensors system (Aquanex system, Swimming Technology Research, Richmond, VA, USA) and defined as a kinetic variable, while speed, stroke rate, stroke length, and stroke index were retrieved and considered as kinematic measures. The top-tier swimmers were taller with a longer arm span and hand surface areas than the low-tier, but similar to the mid-tier. While the mean peak force, speed and efficiency differed among tiers, the stroke rate and stroke length showed mixed findings. Coaches should be aware that young swimmers belonging to the same age group may deliver different performance outcomes due to different kinetic and kinematic behaviors.

## 1. Introduction

From childhood to adulthood, competitive swimming aims to accomplish a given distance in the shortest time [1]. Identifying parameters related to anthropometrics, kinematics, efficiency, hydrodynamics, and energetics plays a key role in swimming performance enhancement [2]. As swimmers’ capacity to move through the water depends on the amount of applied propulsion and resistive forces as opposed to the forward motion, kinetic parameters should also be deeply tested [3]. In front crawl, swimmers propel themselves forward mainly using arm-pulling actions [4] as a consequence of muscular contractions due to biochemical energy production [5]. It is noteworthy that hands have paramount importance to overall propulsion among the hands, upper arm, and forearm [6,7]. Recent research highlighted that a greater amount of applied in-water force by hands leads to higher hand speed [8] and better performance [9]. 

The complexity of unsteady flow mechanics in human swimming imposes a sluggish advance in the knowledge of propulsion mechanics. Still, some progress was made based on the indirect and direct assessment methods [3]. Getting swift and real-time feedback in ecologically valid environments helps researchers and swimming coaches track in-water forces. Thus, a differential pressure system composed of two hand sensors has been proposed as a reliable method to estimate the resultant force of both hands [10] without imposing any constraints on young swimmers’ mechanics or efficiency [11]. However, a paucity of information remains about the understanding of young swimmers’ forces retrieved directly as most of the swimmers tested were above 15 years of age [3,12].

Young swimmers’ competition relies on age groups categorized in chronological age and sex. Although swimmers’ performance depends on a multifactorial and dynamic phenomenon, growth spurts and biological maturation within the same competition age group might affect the swimmer’s expertise level. That is, chronological age and biological age could affect performance as the maturation stage can be several years apart [13]. Indeed, young swimmers’ performance is highly related to anthropometric [14] and biomechanic (50–60%) features [15]. Therefore, the interplay among several parameters could impact swim speed [16] being trigged by different expertise levels [17]. For instance, faster swimmers at younger ages tend to present greater anthropometric traits and optimized stroke mechanics (SR–SL relationship), translating into better swim efficiency and a lower intracycle velocity variation compared to their less skilled counterparts [16,17,18]. However, just one study grouped mature swimmers and analyzed peak and mean in-water forces with pressure sensors [19]. Thus, it remains unanswered if swimmers’ expertise within the same young age group (defined by tiers) leads to different in-water kinetic and kinematic behaviors.

The overall trend of an age group team is usually considered in the swimming performance analysis. However, as performance levels may differ between two swimmers with the same chronological age, grouping them according to their performance (swim speed) could be a more accurate approach and provide deeper insights into the swimmers’ customized path to achieve certain expertise level. The aim of this study was to compare swimmers’ anthropometrics, in-water kinetic, and kinematic behaviors according to different swimming performance tiers (top-, mid-, and low-tier) within the same age group. It was hypothesized that swimmers’ tiers would exhibit different kinetic and kinematic behaviors due to expertise level.

## 2. Materials and Methods

### 2.1. Subjects

In total, 53 highly trained [20] swimmers (girls and boys: 12.40 ± 0.74 years; Tanner stages 1–2) volunteered to participate in this study. Swimmers were recruited from local swimming squads and assessed at the end of the third macrocycle (competitive peak form). The inclusion criteria for the participants were: (i) having a minimum of two years in competitive swimming in regional or national competitions; (ii) practicing in more than four swim training sessions per week; (iii) being previously familiar with the hand differential pressure system; and (iv) not having suffered any injuries in the past six months. All swimmers were ranked based on their personal best performance (i.e., speed) in the 50 m freestyle event (short-course) and then split up into 3 tiers (T): Tier-1, lower-tier (1.25 ± 0.08 m·s^−1^); Tier-2, mid-tier (1.45 ± 0.04 m·s^−1^); and Tier-3, top-tier (1.60 ± 0.04 m·s^−1^). The official race times were obtained from an open-access website (swimrankings.net), and the time gap between the event and the testing sessions did not exceed more than two weeks.

The swimmers’ parents or guardians, as well as the swimmers, were informed about the benefits and experimental risks before signing a written informed consent form. All procedures were in accordance with the Declaration of Helsinki and approved by the Institutional Ethics Committee of the University of Beira Interior (code: CEUBI-Pj-2020-058).

### 2.2. Anthropometrics

Swimmers were measured while wearing a regular textile swimsuit and a cap. The body mass, stature, and arm span were measured by a single researcher following recommended and standardized protocols [21]. Stature and arm span were measured to the nearest 0.1 cm using a portable stadiometer (SECA, 242, Hamburg, Germany) and flexible tape (RossCraft, Surrey, BC, Canada), and body mass was measured to the nearest 0.1 kg using a portable scale (TANITA, BC-730, Amsterdam, Netherlands). The hand surface area (HSA, in cm^2^) for the dominant and non-dominant sides was measured by digital photogrammetry [22]. Swimmers placed each hand on a flat surface with a 2D calibration frame (3 × 3 cm); from there, all images were exported to an on-screen digitizer (Universal Desktop Ruler, v3.8, AVPSoft, Pittsburgh, PA, USA). The swimmers’ hand dominance and Tanner stage were obtained by self-report.

### 2.3. Stroke Kinetics

A standardized warm-up (400 m swim, 100 m pull, 100 kick, 4 × 50 m at increasing speed, 200 m easy swim) was performed individually before the in-water testing. In-water kinetics was measured during the 2 × 25 m front crawl swimming (maximum bouts with a push-off start) using a differential pressure system composed of two hand sensors (Type A, Swimming Technology Research, Richmond, VA, USA). The Aquanex system has been proposed as a reliable method to measure pressure differences between the palmar and dorsal surfaces of both hands [10] without imposing any constraints on the mechanics or efficiency of young swimmers [11]. Sensors were attached by a cable to a two-channel A/D converter connected to a laptop with the Aquanex software (v.4.1, Model DU2, Swimming Technology Research, Richmond, VA, USA). The system was carried with elastic straps on swimmers’ shoulders and arms. The calibration was done as reported elsewhere [9], and data were acquired with a sampling frequency of 100 Hz.

Hand resultant force (in N) was derived from the product of differential pressures by the HSA of each swimmer. Theforce–time curves of underwater paths were analyzed between the 11th and 24th m. The mean peak force (F_PEAK_, in N) of the dominant and non-dominant hand was defined as the mean of the maximum values (i.e., peak) obtained in all stroke cycles of the defined interval. Two visual marks were applied in the defined interval, and the distance covered by the swimmers was recorded using two video cameras (Sony, HDR-CX 240, Tokyo, Japan) at a sampling frequency of 50 Hz. Force data were imported into a signal-processing software (AcqKnowledge v.3.7.3, Biopac Systems, Santa Barbara, CA, USA), and the signal was handled with a 5 Hz cut-off low-pass fourth-order Butterworth filter. The best bout was used for further analysis.

### 2.4. Performance and Stroke Kinematics

In-water temporal and kinematic parameters were retrieved concurrently with the in-water kinetics. The time spent to cover 25 m (T25, in s) was manually assessed using a stopwatch (FINIS 3 × 100, Finis Inc., Livermore, CA, USA). Then, swimming speed (s25, in m·s^−1^) was calculated based on the ratio between the distance of 25 m and the T25 and considered a performance outcome. The stroke rate (SR, in Hz) was assessed with a chrono-frequency meter (FINIS 3 × 300, Finis Inc., Livermore, CA, USA) from three consecutive stroke cycles between the 11^th^ and the 24^th^ m, and the stroke length (SL, in m) was estimated (SL = speed/SR). The SL was also normalized to the arm span (SL/arm span, dimensionless). The stroke efficiency during the 25 m front crawl was estimated using the stroke index (in m^2^·s^−1^) (Equation (1)).

SI = SL × v(1)

where SI (in m^2^·s^−1^) is the stroke index, SL (in m) is the stroke length, and v (in m·s^−1^) is the swimming speed.

### 2.5. Statistical Analysis

The Shapiro–Wilk test was used to assess the normality of data. The mean plus one standard deviation (M ± 1SD) and 95% confidence interval (95CI) were reported for all variables. An unpaired *t*-test was used in each variable to compare differences between girls and boys. The one-way analysis of variance (ANOVA) followed by Tukey’s honest significance difference (HSD) post hoc test was used to analyze differences between each tier in all dependent variables. Partial eta squared (η_p_^2^) was considered as an effect size (ES) measure for ANOVA and interpreted as reported elsewhere [23]: no effect if 0 > |η_p_^2^| ≤ 0.04; a minimum effect if 0.04 > |η_p_^2^| ≤ 0.25; a moderate effect if 0.25 > |η_p_^2^| ≤ 0.64; and a strong effect if |η_p_^2^| > 0.64. Cohen’s d was selected as an effect size (d) for Tukey HSD post hoc and interpreted as: trivial if |d| < 0.2, medium if 0.2 > |d| < 0.5, and large if |d| ≥ 0.5 [24]. The statistical significance was set at *p* ≤ 0.05.

## 3. Results

Boys and girls were pooled and analyzed together as they did not differ in any variable (*p* > 0.05).

### 3.1. Anthropometrics

The effect of swimmers’ tiers in anthropometrics is shown in Table 1. A moderate tier effect was found in stature, arm span, and HSA for both limbs (*p* ≤ 0.05), but no effect was found in body mass. Top-tier (T3) swimmers were taller with a longer arm span and hand surface area than the low-tier (T1) but similar to the mid-tier (T2) in all variables. A large effect was found in all tier comparisons.

### 3.2. Stroke Kinetics

Table 2 demonstrates the effect of swimmers’ tiers in the in-water force. A moderate effect was shown in F_PEAK_ D and ND. Low-tier swimmers delivered a lower amount of force in both limbs, while top-tier swimmers showed higher values. However, no differences were found between mid- and top-tier swimmers for F_PEAK_ D. Despite this, a large effect was found between all tiers.

### 3.3. Performance and Stroke Kinematics

Table 3 shows the effect of swimmers’ tiers on performance and stroke kinematics. The top-tier swimmers were the ones with the higher s25. The SR and SL showed mixed findings. While the top- and mid-tier swimmers had the same SL, low-tier swimmers exhibited a shorter SL. The SR was similar for low- and mid-tier swimmers, while the top-tier were able to deliver a higher SR. A large effect was found between all tiers.

## 4. Discussion

The present study aimed to compare swimmers’ anthropometrics and in-water kinetic and kinematic behaviors according to different swimming performance tiers within the same age group. The main results indicated large effects for most parameters when comparison was done among the tiers. Top-tier swimmers were shown to be taller with long limbs and higher in-water force, along with better stroke mechanics and efficiency than low-tier swimmers. However, mid-tier and top-tier swimmers revealed a similar anthropometric profile with mixed findings for in-water kinetic and kinematic parameters.

### 4.1. Anthropometrics

Anthropometric features are highly associated with young swimmers’ performance [14,25], and those with larger body traits are more likely to swim faster [17,26]. However, natural growth and biological maturation are underlying processes during youth that may change any performance outcome [27]. For instance, maturation takes the lead in defining the anthropometric changes after a training cessation of young swimmers [22,28]. Moreover, growth spurts experienced at different moments lead to high inter-swimmer variability within the same age group [18]. The findings of the present study showed that swimmers ranked in the top-tier are more likely to demonstrate large body traits than the low-tier, whereas a similar profile was observed between the mid-tier and top-tier. Recent studies found similar anthropometric differences between the top-tier and low-tier in young swimmers of the same age group [17,29]. Contrarily, our results demonstrated that low-tier and mid-tier differ only in the HSA of both limbs. It can be argued that swimmers’ maturation interplay within the same age group with the mid- and top-tier presents a similar status at the point of assessment. Furthermore, it seems that spurt in stature or length are linked to the later stages of development, whereas hands and feet experience accelerated growth during the early stages [30]. As swimmers were categorized in Tanner stages 1–2, a deep understanding of maturity status among tiers is limited. Moreover, body mass was similar among tiers, which can be related to lean body mass or body fat mass leading to better or worse performances, respectively. Therefore, future studies should try to understand the maturity offset within the same age-group and according to each performance tier. Nevertheless, coaches and researchers should be aware that different anthropometric profiles are shown within the same age group, and swimmers seem to shift their level of expertise (i.e., from one tier to another) within and between competitive seasons [29].

### 4.2. Stroke Kinetics

In-water force may determine the overall stroke mechanics and then influence performance [2]. At least for adult swimmers, a greater propulsive force acting in the propulsive direction leads to a better performance in a 20 m front crawl [8]. Additionally, the expertise level seems to be a factor that influences the hand propulsive force [31], as well as the hand resultant force [19] in adult/mature swimmers. Due to the contribution to overall performance, some progress has been made in measuring in-water forces during free motion (i.e., “free swimming”) and using straightforward and user-friendly apparatus [3]. Despite this, some of those systems only measure hand resultant force instead of effective propulsive force [10].

Although the use of pressure sensors has been growing in interest, studies with young swimmers are still few. The force values in the present study align with those from the previous studies using young swimmers [10,28]. Adult swimmers are expected to exert a higher amount of force [12], but some studies showed similar or lower values when testing older swimmers with the same setup of sensors, e.g., [19]. It can be argued that a different methodology to analyze data was employed in previous studies, and therefore, a careful comparison of hand resultant force behavior needs to be made.

As far as our understanding goes, no study aimed to gather young swimmers within the same age group to analyze the behavior of in-water forces according to their level of expertise. In this study, a moderate group effect was found for F_PEAK_ of both limbs. Swimmers with better performances (top-tier) showed higher F_PEAK_ (approx. 64–65 N), while the opposite was found for the low-tier (approx. 41–45 N). Although no differences were found between the D between mid-tier and top-tier, it seems that different force behaviors should be expected according to different tiers. The previous literature on the topic reports that in-water force is a key factor in distinguishing swimmers with better and worst performances [19,31]. A higher competitive level was associated with a greater hand propulsive force [31], as the faster the hands move, the greater the hydrodynamic force exerted by the hand [8]. An increase in SR could also increase swimming speed and hand propulsive force [9], with an optimal angle of attack being considered important to maintain the hand propulsive force [8]. Such rationale might explain the differences between tiers, as changes in motor control due to growth may influence stroke kinematics and kinetics in young swimmers. Santos et al. [28] found that in-water kinetics are directly related to the maturity offset of swimmers within the same age group. Overall, in-water force can be deemed as an important parameter for swimmers’ excelling (i.e., better performance) at these early ages.

### 4.3. Performance and Stroke Kinematics

Swimming speed depends on the relationship between SR and SL, and an increase can be reached using different individual SR–SL combinations [2]. An increase in SF seems to lead to a higher energy cost [32], whereas increasing SL is associated with a more economical swim in front crawl [33]. A long SL has been pointed out as paramount in young swimmers (e.g., [26]). Although a long SL seems to be related to a long arm span [34], improving SR while maintaining SL is a challenge at early ages [22].

While the faster swimmers (i.e., top-tiers) presented a long SL with a higher SR, the slower swimmers (low-tier) showed a short SL along with a lower SR. A long SL but a lower SR was the trend for the mid-tier swimmers. The present results are corroborated by a recent study that found the same stroke mechanics profile for three tiers within the same age group [17]. The same authors argued that low-tier does not excel due to the lack of “nature” (i.e., anthropometrics) and “nurture” (i.e., swimming biomechanics and energetics) characteristics. Still, such “nature” disadvantages can be overcome by individual adaptations in stroke mechanics [16], i.e., with an optimal individual SR–SL relationship. Despite this, all tiers differed in 25 m front crawl performance, agreeing with the previous studies conducted on young [16,17] and mature swimmers [19].

The SL/arm span ratio was similar independently of swimmers’ tier. While Barbosa et al. [17] noted differences between the low-tier and the remaining two tiers, Figueiredo et al. [16] reported no differences in the ratio among the three groups. Moreover, SI is an efficiency proxy based on the swimming speed and SL, and thus, if a given speed is considered, swimmers are more efficient with a longer SL. Therefore, one can argue that the SI of mid-tier and top-tier were similar due to the similar SL, but less efficiency is linked to a worse SL in the low-tier. Accordingly, coaches should seek to constantly monitor stroke mechanics (i.e., technique) in their young swimmers in order to better interpret how they are positioned in the different tiers that exist in a single swimming squad.

## 5. Conclusions

Performance tiers within the same competitive age group of young swimmers demonstrate different in-water kinetic and kinematic behaviors. Top-tier swimmers seem to apply a greater amount of force accompanied by better stroke mechanics than low-tier swimmers. Thus, exploring theinter–individual profiles at younger ages may help coaches to group swimmers properly and design the most suitable training for further improvement.

## Figures and Tables

**Table 1:** Descriptive analysis and effect of tiers in swimmers’ anthropometrics.

Dependent Variable	Tiers			One-Way ANOVA			Post Hoc		
	T1 M ± SD (95CI)	T2 M ± SD (95CI)	T3 M ± SD (95CI)	F-Value	*p*-Value	ES	T1–T2 *p* (d)	T2–T3 *p* (d)	T1–T3 *p* (d)
Body mass (kg)	46.3 ± 9.0 (41.3–51.2)	48.4 ± 8.9 (43.7–53.2)	51.1 ± 8.0 (46.8–55.4)	1.233	0.301	0.05	0.759 (0.24)	0.661 (0.33)	0.271 (0.58)
Stature (cm)	152.7 ± 5.9 (149.4–156.0)	157.1 ± 6.1 (153.9–160.4)	161.5 ± 7.5 (157.5–165.6)	6.933	0.002	0.25	0.160 (0.75)	0.155 (0.66)	0.002 (1.35)
Arm span (cm)	152.1 ± 7.3 (148.1–156.2)	157.2 ± 6.9 (153.5–160.9)	163.2 ± 9.1 (158.4–168.1)	7.788	0.001	0.26	0.183 (0.74)	0.086 (0.77)	<0.001 (1.39)
HSA D (cm^2^)	94.8 ± 8.1 (90.6–98.9)	104.5 ± 11.2 (98.9–110.1)	111.7 ± 9.9 (106.4–116.9)	12.175	<0.001	0.34	0.015 (1.02)	0.100 (0.70)	<0.001 (1.93)
HSA ND (cm^2^)	94.8 ± 8.5 (90.4–99.2)	104.9 ± 11.2 (99.4–110.5)	112.0 ± 10.2 (106.6–117.5)	12.190	<0.001	0.34	0.013 (1.05)	0.112 (0.68)	<0.001 (1.88)

D, dominant; ES, effect size; HSA, hand surface area; ND, non-dominant; T, tier; T1, lower-tier; T2, mid-tier; T3, top-tier.

**Table 2:** Descriptive analysis and effect of tiers in swimmers’ in-water force.

Dependent Variable	Tiers			One-Way ANOVA			Post-Hoc		
	T1 M ± SD (95CI)	T2 M ± SD (95CI)	T3 M ± SD (95CI)	F-Value	*p*-Value	ES	T1–T2 *p* (d)	T2–T3 *p* (d)	T1–T3 *p* (d)
F_PEAK_ D (N)	44.5 ± 8.6 (40.2–48.7)	56.3 ± 10.9 (50.9–61.7)	65.1 ± 14.1 (57.8–72.3)	14.548	<0.001	0.37	0.008 (1.24)	0.069 (0.72)	<0.001 (1.83)
F_PEAK_ ND (N)	41.3 ± 11.7 (35.4–47.1)	54.1 ± 9.8 (49.2–58.9)	63.9 ± 13.6 (56.9–70.9)	16.295	<0.001	0.40	0.006 (1.22)	0.044 (0.86)	<0.001 (1.84)

D, dominant; ES, effect size; F_PEAK_, mean peak force; ND, non-dominant; T, tier; T1, lower-tier; T2, mid-tier; T3, top-tier.

**Table 3:** Descriptive analysis and effect of tiers in swimmers’ performance and in-water kinematics.

Dependent Variable	Tiers			One-Way ANOVA			Post Hoc		
	T1 M ± SD (95CI)	T2 M ± SD (95CI)	T3 M ± SD (95CI)	F-Value	*p*-Value	ES	T1–T2 *p* (d)	T2–T3 *p* (d)	T1–T3 *p* (d)
s25 (m·s^−1^)	1.32 ± 0.08 (1.28–1.36)	1.47 ± 0.05 (1.45–1.49)	1.58 ± 0.06 (1.55–1.61)	70.864	<0.001	0.74	<0.001 (2.31)	<0.001 (2.06)	<0.001 (3.77)
SR (Hz)	0.76 ± 0.07 (0.72–0.79)	0.77 ± 0.07 (0.73–0.80)	0.83 ± 0.07 (0.80–0.86)	5.480	0.007	0.18	0.870 (0.15)	0.032 (0.88)	0.009 (1.03)
SL (m)	1.75 ± 0.14 (1.68–1.82)	1.93 ± 0.18 (1.84–2.01)	1.91 ± 0.13 (1.84–1.98)	7.112	0.002	0.22	0.004 (1.15)	0.968 (0.13)	0.009 (1.22)
SL/Arm span	1.16 ± 0.09 (1.10–1.21)	1.21 ± 0.11 (1.15–1.28)	1.17 ± 0.08 (1.13–1.22)	1.334	0.274	0.06	0.287 (0.51)	0.438 (0.43)	0.940 (0.12)
SI (m^2^·s^−1^)	2.31 ± 0.27 (2.18–2.45)	2.83 ± 0.28 (2.69–2.97)	3.02 ± 0.25 (2.89–3.15)	32.764	<0.001	0.57	<0.001 (1.95)	0.102 (0.74)	<0.001 (2.81)

ES, effect size; s25, swimming speed; SI, stroke index; SL, stroke length; SR, stroke rate; T, tier; T1, lower-tier; T2, mid-tier; T3, top-tier.

## Data Availability

The data presented in this study are available on request from the corresponding author.
